# Unspecific reactivity must be excluded in COVID-19 epidemiological analyses or virus tracing based on serologic testing: Analysis of 46,777 post-pandemic samples and 1,114 pre-pandemic samples

**DOI:** 10.3389/fmed.2022.1018578

**Published:** 2022-11-16

**Authors:** Min-Jing Cai, Jie Lin, Jian-Hui Zhu, Zhang Dai, Yi-Qiang Lin, Xian-Ming Liang

**Affiliations:** ^1^Centre of Clinical Laboratory, Zhongshan Hospital of Xiamen University, School of Medicine, Xiamen University, Xiamen, China; ^2^Institute of Infectious Disease, School of Medicine, Xiamen University, Xiamen, China

**Keywords:** SARS-CoV-2, serologic testing, unspecific reactivity, antibody, COVID-19

## Abstract

**Background:**

Severe acute respiratory syndrome coronavirus 2 (SARS-CoV-2) is the causative agent of coronavirus disease 2019 (COVID-19). Serologic testing is complementary to nucleic acid screening to identify SARS-CoV-2. This study aimed to evaluate unspecific reactivity in SARS-CoV-2 serologic tests.

**Materials and methods:**

Total anti-SARS-CoV-2 antibodies from 46,777 subjects who were screened for SARS-CoV-2 were retrospectively studied to evaluate the incidence and characteristics of the unspecific reactivity. A total of 1,114 pre-pandemic samples were also analysed to compare unspecific reactivity.

**Results:**

The incidence of unspecific reactivity in anti-SARS-CoV-2 total antibody testing was 0.361% in 46,777 post-pandemic samples, similar to the incidence of 0.359% (4/1,114) in 1,114 pre-pandemic samples (*p* = 0.990). Subjects ≥ 19 years old had a 2.753-fold [95% confidence interval (CI), 1.130–6.706] higher probability of unspecific reactivity than subjects < 19 years old (*p* = 0.026). There was no significant difference between the sexes. The unspecific reactivity was associated with 14 categories within the disease spectrum, with three tops being the skin and subcutaneous tissue diseases (0.93%), respiratory system diseases (0.78%) and neoplasms diseases (0.76%). The percentage of patients with a titer ≥ 13.87 cut-off index (COI) in the unspecific reactivity was 7.69%.

**Conclusion:**

Our results suggest a unspecific reactivity incidence rate of 0.361% involving 14 categories on the disease spectrum. Unspecific reactivity needs to be excluded when performing serologic antibody testing in COVID-19 epidemiological analyses or virus tracing.

## Introduction

SARS-CoV-2 is the causative agent of coronavirus disease 2019 (COVID-19), which caused a pandemic due to its rapid transmission and strong infectivity (1). The global epidemiological situation of COVID-19 remains serious. A rapid and accurate diagnosis is key in controlling the spread of the disease (2, 3). Serologic testing is complementary to nucleic acid screening for the identification of SARS-CoV-2 (4–6). As the pandemic developed, serologic testing was used to evaluate the effectiveness of vaccination and the prevalence of SARS-CoV-2 infection (7, 8). In addition, serologic tests have been employed to trace SARS-CoV-2 (9, 10). SARS-CoV-2 encodes four structural proteins, namely, the spike (S), envelope (E), membrane (M), and nucleocapsid (N) proteins, among which the spike and nucleocapsid proteins are most commonly detected in SARS-CoV-2 serologic assays (11). One hundred percent cross-reactivity with the full-length SARS nucleocapsid protein has been reported, suggesting that there are polyreactive antibodies in the natural immunoglobulin repertoire with affinity toward some epitopes shared by coronaviruses (12). However, no cross-reactivity in any healthy serum samples with the full-length SARS spike protein has been reported (12). The SARS-CoV-2 spike protein shares 76% homology with that of SARS-CoV-1 and only approximately 30% homology with those of seasonal Beta-CoVs (13). When testing for the presence of SARS-CoV-2 antibodies, researchers have utilized the full spike ectodomain as well as the receptor-binding domain (RBD) for antigens detection in serologic assays (14). However, this produces an unspecific reactivity, which causes difficulties in clinical diagnosis and treatment (1, 15, 16). Currently, the incidence, correlation factor and characteristics of unspecific reactivity in SARS-CoV-2 serologic tests based on RBD antigens was unclear. Investigating unspecific reactivity will greatly benefit serological diagnosis, epidemiological investigation, control of SARS-CoV-2 and even virus traceability (9, 17). Here, we retrospectively analysed samples from 46,777 subjects who were screened for identify SARS-CoV-2 infection to investigate the incidence and characteristics of the unspecific reactivity. For comparison, we also investigated the unspecific reactivity in 1,114 pre-pandemic samples.

## Materials and methods

### Study design and participants

**FIGURE 1:**
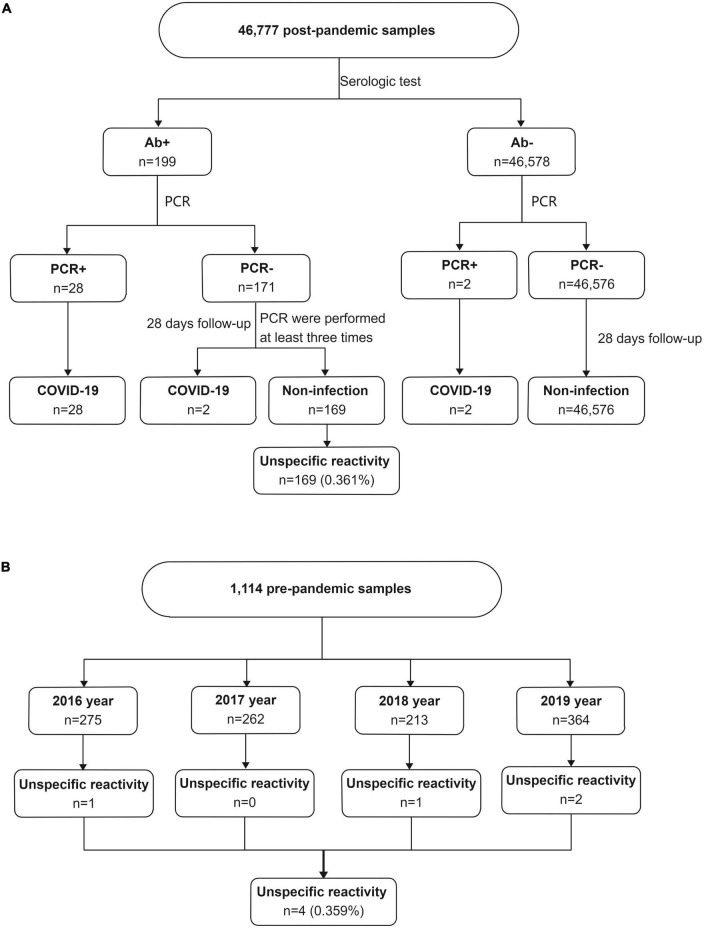
The unspecific reactivity in anti-SARS-CoV-2 total antibody testing. **(A)** The unspecific reactivity in 46,777 post-pandemic subjects who were screened for SARS-CoV-2 infection, **(B)** the unspecific reactivity in 1,114 pre-pandemic frozen serum samples. Ab, total anti-SARS-CoV-2 antibody; PCR, polymerase chain reaction.

Consecutive patients who were screened for SARS-CoV-2 infection by serologic tests and RT-PCR between March 2020 and November 2021 in Zhongshan Hospital were retrospectively evaluated. Zhongshan Hospital is a large integrated Grade III-A hospital that provides approximately 2.50 million people with health care and outpatient medical and hospital services each year. A total of 46,777 subjects without vaccination were screened to identify SARS-CoV-2 infection based on epidemiological history, clinical symptoms, imaging findings and laboratory test results (Figure 1). All subjects underwent anti-SARS-CoV-2 total antibody (Ab) and PCR testing to identify SARS-CoV-2 infection. Those who were Ab-/PCR + and Ab + /PCR + were escorted directly to the hospital for a comprehensive evaluation and epidemiological investigation. Follow-up was performed 28 days later for PCR-/Ab- individuals. PCR-/Ab + individuals were assigned to a key screening population who were followed for 28 days and underwent multiple rounds of PCR testing during follow-up. The subjects with COVID-19 included asymptomatic COVID-19, symptomatic COVID-19 and convalescent patients. Subjects with unspecific reactivity subjects were the no infection subjects with positive serological test results. In unspecific reactivity subjects, PCR was performed at least three times despite negative results during a follow-up period of 28 days.

To investigate the unspecific reactivity before the date of the first COVID-19 case, total antibodies were measured in 1,114 frozen serum samples collected from January 2016 to July 2019 at the Center of Clinical Laboratory, Zhongshan Hospital. Based on the timeline of the first COVID-19 case and the clinical and follow-up data, a positive reaction in a frozen serum sample was considered an unspecific reactivity. Disease classification was based on the International Classification of Diseases, Revision 10 (ICD-10).

### Serologic testing

Blood samples were centrifuged at 3,000 × g, and the upper serum layer was used for testing. The Wantai^®^Caris 200 system (a closed and fully automatic system) was used to measure the total SARS-CoV-2 antibody titer. The detection experiments were performed using a kit from Wantai (Biological Pharmacy Enterprise Co., Ltd., Beijing, China) using a chemiluminescence microparticle immunoassay (CMIA) instrument (Wantai Biological Pharmacy Enterprise Co., Ltd., Beijing, China). Total antibody detection was based on a double-antigen sandwich immunoassay using two kinds of mammalian cell-expressed recombinant antigens containing the receptor-binding domain (RBD) of the spike protein of SARS-CoV-2 as the immobilized antigen and 2′,6′-dimethyl-4′-(*N*-succinimidyloxycarbonyl)phenyl-10-methyl-acridinium-9-carboxylate-1-propanesulfonate inner salt (NSP-DMAE-NHS)-conjugated antigens. The antibody titer was calculated according to the signal to cut-off ratio and was recorded as the cut-off index (COI): a COI < 1.00 was considered negative, and a COI ≥ 1.00 was considered positive.

### Polymerase chain reaction assays for SARS-CoV-2

Upper respiratory tract samples were collected from both oropharyngeal swabs and nasopharyngeal by medical personnel with regularity trained. For lower respiratory tract specimens, participants were given instructions the night before to collect first morning sputum samples (after gargling) in a specimen cup. The Allplex 2020-nCoV assay (Seegene, Seoul, South Korea) was used to perform PCR assays to detect SARS-CoV-2 infection *via* the identification of three genetic markers. These three genetic markers were the envelope (env) gene, RNA-dependent RNA polymerase (RdRp) gene, and nucleocapsid protein (N) gene. The cycle threshold (Ct) determined during RT-PCR testing refers to the cycle in which the detection of viral amplicons occurs, and it is inversely correlated with the amount of RNA present. When the cycle threshold values of all genes were less than 40 cycles, the results were considered positive. Double-site positives or two consecutive single-site positives were judged to indicate RT-PCR positivity according to the COVID-2019 Prevention and Control Plan (Eighth Edition).

### Statistical analysis

Statistical analysis was conducted using SPSS 26.0 (SPSS, Inc., Chicago, IL, USA) and GraphPad Prism version 8.0 (GraphPad Software, San Diego, CA, USA). Continuous variables that did not follow a normal distribution are reported as medians with interquartile ranges (IQRs). The Pearson χ^2^ test was used for analysis of the unspecific reactivity rate. The Mann-Whitney U test was applied for group comparisons. Factors were entered into a logistic regression model. A receiver operator characteristic curve (ROC) was constructed to analyse the total antibody titers in the unspecific reactivity and the COVID-19. Sensitivity and specificity were calculated from the ROC curve. The threshold for significance was a *p*-value < 0.05.

## Results

### Characteristics of unspecific reactivity

A total of 46,777 subjects without vaccination were investigated. Of them, 169 subjects had unspecific reactivity (Figure 1A). The incidence of the unspecific reactivity in the anti-SARS-CoV-2 total antibody was 0.361% (169/46,777). Logistic regression was used to analyse the effects of sex and age in the unspecific reactivity group, with no significance different between the sexes. Subjects ≥ 19 years old had a 2.753-fold (95% CI, 1.130–6.706) higher probability of a unspecific reactivity than subjects < 19 years old (*p* = 0.026) (Table 1).

**TABLE 1:** Factors associated with the occurrence of unspecific reactivity.

Risk factor	No infection	Unspecific reactivity (%)	OR	OR (95% CI)		*P*
					
				Lower	Upper	
Sex						
Female	23,597	83 (0.35)	1			
Male	23,148	86 (0.37)	0.947	0.700	1.280	0.722
Age						
<19	3,611	5 (0.14)	1			
≥19	43,134	164 (0.38)	2.753	1.130	6.706	0.026

A total of 1,114 frozen blood specimens were used to investigate the unspecific reactivity before the date of the first COVID-19 case. Based on the date of the first COVID-19 case, clinical data and the results of telephone follow-up, a positive reaction was considered a unspecific reactivity. The unspecific reactivity rate before the date of the first COVID-19 case was 0.359% (4/1,114) (Figure 1B), which was similar to that during the COVID-19 epidemic (*p* = 0.990). Patients with unspecific reactivity before the first COVID-19 case were diagnosed with ulcerative colitis, fever, systemic lupus erythematosus and testicular tumor.

### Disease spectrum of unspecific reactivity

A total of 169 cases were classified into 14 categories within the disease spectrum according to the ICD-10. Among those with unspecific reactivity, the top three categories were diseases of the skin and subcutaneous tissue, respiratory system diseases and neoplasms, with incidence rates of 0.93%, 0.78%, and 0.76%, respectively. Among those with unspecific reactivity, diseases with proportions between 0.60%–0.70% were diseases of the eye and adnexa, the digestive system and the nervous system. Diseases with proportions between 0.40%–0.59% were diseases of the blood and blood-forming organs and certain disorders involving the immune mechanism, diseases of the genitourinary system and diseases of the musculoskeletal system and connective tissue (Table 2).

**TABLE 2:** Disease spectrum associated with unspecific reactivity according to the International Classification of Diseases, Revision 10.

Sorting	Disease	No. of unspecific reactivity	The unspecific reactivity proportion (%)
1	Diseases of the skin and subcutaneous tissue	3	0.93 (3/322)
2	Diseases of the respiratory system	28	0.78 (28/3,568)
3	Neoplasms	40	0.76 (40/5,240)
4	Diseases of the eye and adnexa	5	0.63 (5/800)
5	Diseases of the digestive system	19	0.60 (19/3,145)
6	Diseases of the nervous system	4	0.60 (4/670)
7	Diseases of the blood and blood-forming organs and certain disorders involving the immune mechanism	3	0.49 (3/607)
8	Diseases of the genitourinary system	13	0.46 (13/2,838)
9	Diseases of the musculoskeletal system and connective tissue	9	0.41 (9/2,173)
10	Endocrine, nutritional and metabolic diseases	3	0.37 (3/802)
11	Diseases of the circulatory system	11	0.37 (11/2,947)
12	Pregnancy, childbirth, and puerperium	6	0.34 (6/1,759)
13	Injury, poisoning and certain other consequences of external causes	5	0.33 (5/1,505)
14	Symptoms, signs and abnormal clinical and laboratory findings not elsewhere classified	20	0.31 (20/6,514)
15	Other	0	0.00 (0/13,855)

### Unspecific reactivity titer

**FIGURE 2:**
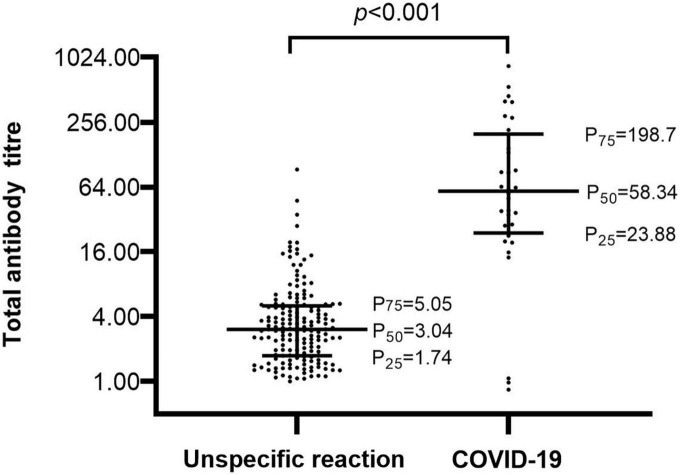
Comparison of total antibody titers between the unspecific reactivity and COVID-19. Bars represent the arithmetic median with the interquartile range.

The titer of the unspecific reactivity group was 3.04 (1.74–5.05) COI, which was significantly lower than 58.34 (23.88–198.7) COI in the COVID-19 group (*p* < 0.001) (Figure 2). In the receiver operating curve (ROC) analyses of total antibody titers in unspecific reactivity and COVID-19 groups, the cut-off value was 13.87 COI, with 90.63% (95% CI: 73.83–97.55%) sensitivity and 92.31% (95% CI: 86.93–95.67%) specificity (Table 3). The number of cases with titer ≥ 13.87 COI in unspecific reactivity was only 13 (7.69%) (Table 4). Among the 1,114 frozen blood specimens, all of the titers in unspecific reactivity were less than 13.87 COI.

**TABLE 3:** Diagnostic efficacy of the total antibody titer in unspecific reactivity and COVID-19 according to the cut-off of 13.87 cut-off index (COI).

		COVID-19		Sensitivity (%) (95% CI)	Specificity (%) (95% CI)
				
		+	−		
Total antibody	+	29	13	90.63	92.31
	–	3	156	(73.83–97.55)	(86.93–95.67)

**TABLE 4:** Disease spectrum in unspecific reactivity with a total antibody titer ≥ 13.87 cut-off index (COI).

Disease	No (%)	Antibody titer (COI)
Neoplasms	3 (23.10%)	14.81, 47.70, 93.20
Diseases of the skin and subcutaneous tissue	2 (15.38%)	19.47, 35.40
Diseases of the respiratory system	2 (15.38%)	15.38, 15.40
Diseases of the digestive system	2 (15.38%)	14.35, 19.59
Diseases of the musculoskeletal system and connective tissue	1 (7.69%)	27.84
Diseases of the nervous system	1 (7.69%)	17.84
Diseases of the circulatory system	1 (7.69%)	16.33
Pregnancy, childbirth and puerperium	1 (7.69%)	16.90
Total	13	

## Discussion

Serologic testing is a complementary to nucleic acid screening for the identification of SARS-CoV-2 infection (4–6). Currently, the incidence, correlative factors and characteristics of unspecific reactivity in SARS-CoV-2 serologic tests based on RBD antigens are unclear. In our study, 46,777 subjects were retrospectively investigated between March 2020 and November 2021. The incidence of unspecific reactivity in the anti-SARS-CoV-2 total antibody test was 0.361%, which was similar to the incidence of 0.359% among 1,114 blood specimens collected before the first COVID-19 case. Subjects ≥ 19 years old had a 2.753-fold (95% CI, 1.130–6.706) higher probability of a unspecific reactivity than subjects < 19 years old. There was no significance different between the sexes. The unspecific reactivity was associated with 14 categories within the disease spectrum. The three top categories were diseases of the skin and subcutaneous tissue, respiratory system and neoplasms. The percentage of subjects with titer ≥ 13.87 COI in the unspecific group was 7.69%.

Serologic testing has been used to elucidate the timeline of the COVID-19 pandemic. Some researchers have used the frozen blood specimens collected before the COVID-19 epidemic to screen for SARS-CoV-2 antibodies to trace the source of SARS-CoV-2 (9, 10). Basavaraju et al. reported that SARS-CoV-2 was present in the United States earlier than previously recognized (9). We used the CMIA double-antigen sandwich method to detect the total antibodies against SARS-CoV-2 in serum and found 169 unspecific reactivity results; with an incidence of 0.361%. Pfluger et al. summarized three automatic serological total antibody detection methods for SARS-CoV-2, and all assays had false positive rates of 0.6% (2/320, ELISA), 0.3% (1/320, ECLIA) and 0.0% (0/320, CLIA) (18). Overall, the incidence rates of unspecific reactivity were low, never exceeding 1%. Furthermore, the unspecific reactivity incidence before the date of the first COVID-19 case was 0.359%, which was similar to 0.361% during the COVID-19 epidemic in our study. This result indicates that the unspecific reactivity are inevitable. The unspecific reactivity could confound the results of the source of SARS-CoV-2 according to tracing based on serologic testing.

The unspecific reactivity tendency has been shown to be higher in older populations with antibodies against other pathogens (19, 20). In this study, the unspecific reactivity was associated with age, with subjects ≥ 19 years having a higher probability. With increasing age, endogenous interfering substances such as rheumatoid introducers (RFs) and cross-antigens in the blood increase, which could affect antibody detection and lead to false-positive results (21, 22). In addition, we investigated the disease spectra of patients with unspecific reactivity and found that the three top categories were diseases of the skin and subcutaneous tissue, respiratory system, and neoplasms. The causes may be as follows. First, a high incidence of unspecific reactivity has been reported in those with diseases of the skin and subcutaneous tissue (19), and these diseases might be associated with potential autoimmune aetiologias that produce abnormal expression of IgE, IgG, or IL-1 (23–26), resulting in a unspecific reactivity (27). Second, a high incidence of non-specific reactivity has been reported in those with diseases of the respiratory system, and an unspecific reactivity in SARS-CoV-2 antibody testing may be due to previous infection with other human coronaviruses (HCoVs); in fact, cross reaction between nucleocapsid or spike proteins of different HCoVs has been reported (28, 29). Finally, neoplasms, the third most common disease category, regularly induce adaptive immune responses in humans and may lead to abnormal protein expression (30, 31). The unspecific reactivity in a serologic test may result from endogenous interfering substances or cross reactivity antibodies. This result supports the notion that unspecific reactivity is unavoidable. Unspecific reactivity needs to be excluded when performing serologic antibody testing in SARS-CoV-2 tracing.

It is worth noting that the titer of unspecific reactivity was much lower than that of the COVID-19. If a risk assessment dictates an overriding concern, the cut-off can be set accordingly (11). In the ROC analysis of the total antibody titer in unspecific reactivity and COVID-19 groups, a cut-off value of 13.87 COI was established, with 90.63% sensitivity and 92.31% specificity. A low titer was one of the characteristics of unspecific reactivity. To exclude the unspecific reactivity, the cut-off value should be revaluated according to the specific objective and population.

This study has some limitations. First, as a retrospective study, endogenous interfering substances in serum, such as RF, heterophile antibodies and cross-antigens, were not detected. Second, the unspecific reactivity in frozen serum were defined based on clinical data and not pathogen detection of SARS-CoV-2. Third, this retrospective study was conducted in a single hospital. Finally, the unspecific reactivity group was not checked according to PCR based on N gene mutations of SARS-CoV-2.

## Conclusion

The incidence rate of unspecific reactivity was 0.361% and was similar to the incidence of 0.359% before the first COVID-19 case. Subjects ≥ 19 years old had a 2.753-fold higher probability of unspecific reactivity than subjects < 19 years old. There were 14 categories within the disease spectrum associated with unspecific reactivity. Titers in unspecific reactivity were generally low (COI < 13.87). Unspecific reactivity needs to be excluded when using serologic antibody testing for COVID-19 epidemiological analysis or virus tracing.

## Data availability statement

The raw data supporting the conclusions of this article will be made available by the authors, without undue reservation.

## Ethics statement

The studies involving human participants were reviewed and approved by Institutional Ethics Committee of Zhongshan Hospital of Xiamen University, School of Medicine, Xiamen University (#xmzsyyky2021196). Written informed consent for participation was not required for this study in accordance with the national legislation and the institutional requirements.

## Author contributions

X-ML: conceptualization, formal analysis, project administration, study design, funding, writing, and manuscript submission. M-JC and JL: data analyses, interpretation, manuscript drafting, and critical revision of the manuscript. J-HZ: data acquisition. ZD and Y-QL: production of tables and figures. All authors contributed to the article and approved the submitted version.
